# Nanoparticle-Based Delivery Systems for Vaccines

**DOI:** 10.3390/vaccines10111946

**Published:** 2022-11-17

**Authors:** Rajashri Bezbaruah, Vivek P. Chavda, Lawandashisha Nongrang, Shahnaz Alom, Kangkan Deka, Tutumoni Kalita, Farak Ali, Bedanta Bhattacharjee, Lalitkumar Vora

**Affiliations:** 1 Department of Pharmaceutical Sciences, Faculty of Science and Engineering, Dibrugarh University, Dibrugarh 786004, Assam, India; 2 Department of Pharmaceutics and Pharmaceutical Technology, L. M. College of Pharmacy, Ahmedabad 380008, Gujarat, India; 3 Department of Pharmacology, Girijananda Chowdhury Institute of Pharmaceutical Science-Tezpur, Sonitpur 784501, Assam, India; 4 Department of Pharmacognosy, NETES Institute of Pharmaceutical Science, Mirza, Guwahati 781125, Assam, India; 5 Department of Pharmaceutical Chemistry, Girijananda Chowdhury Institute of Pharmaceutical Sciences, Azara, Guwahati 781017, Assam, India; 6 Department of Pharmaceutical Chemistry, Girijananda Chowdhury Institute of Pharmaceutical Science-Tezpur, Sonitpur 784501, Assam, India; 7 School of Pharmacy, Queen’s University, Belfast BT9 7BL, UK

**Keywords:** nanoparticles, nanovaccine, nanocarriers, exosome

## Abstract

Vaccination is still the most cost-effective way to combat infectious illnesses. Conventional vaccinations may have low immunogenicity and, in most situations, only provide partial protection. A new class of nanoparticle-based vaccinations has shown considerable promise in addressing the majority of the shortcomings of traditional and subunit vaccines. This is due to recent breakthroughs in chemical and biological engineering, which allow for the exact regulation of nanoparticle size, shape, functionality, and surface characteristics, resulting in improved antigen presentation and robust immunogenicity. A blend of physicochemical, immunological, and toxicological experiments can be used to accurately characterize nanovaccines. This narrative review will provide an overview of the current scenario of the nanovaccine.

## 1. Introduction

Vaccination is the practice of delivering an antigenic substance into a person’s body to activate their immune system and generate adaptive immunity against a pathogen [1]. It has been demonstrated to be the most efficient and cost-effective means of preventing infectious ailments. Many important illnesses, including mumps, tetanus, smallpox, polio, measles, pertussis, rubella, diphtheria, and yellow fever, have been eradicated or well-managed, courtesy of vaccines [2,3]. Notwithstanding these instances of successful vaccinations, many disease conditions, such as tuberculosis, acquired immunodeficiency syndrome (AIDS), dengue fever, and malaria, lack an efficient prophylactic strategy. As a result, the search for innovative vaccination formulations and technologies persists [4]. Vaccine formulations are typically made up of attenuated subunit protein antigens and inactivated microbes that trigger a particular immunological activation. Every system has its unique collection of upsides and downsides, and safety and efficacy are often traded off.

A nanoparticle is defined as a particle with at least one dimension of <100 nm. It is considered to be the building block for nanotechnology. The ability to synthesize and manipulate such materials has led to a recent resurgence in the use of particles in these size ranges by several industries and humans [5]. The use of nanoparticles as nanosystems displaying pertinent antigenic groups as a potential alternative for traditional vaccinations is fascinating [6,7,8]. These nanosized particles can be of natural or synthetic origin [8]. Technological breakthroughs have made it possible to create nanoparticles (NPs) featuring distinctive physicochemical features. Size, solubility, shape, hydrophilicity, and surface chemistry, for example, may all be tweaked and regulated, enabling the creation of NPs with specific biological features [9]. NPs may also be constructed to facilitate the inclusion of a wide array of molecules, such as antigens, making them very useful in vaccine development [10,11]. Antigens can be incorporated into NPs by conjugation (covalent functionalization) or encapsulation (physical trapping) [12,13]. The conjugation of antigens onto NPs allows the immunogen to be exposed to immune systems in a manner similar to how it would be delivered by a virus, prompting a comparable reaction. Antigenic material is encapsulated in NPs, which makes it possible to administer antigens that would otherwise disintegrate quickly or trigger a localized immune response. Furthermore, NPs manufactured from certain composites offer not only site-directed antigen presentation but also antigen release over time to enhance immune system exposure [14,15].

NPs have been found to promote antigen distribution to antigen-presenting cells (APCs) and/or conserve the morphology of antigens against proteolytic cleavage [12,16]. NPs with antigens may also have a local depot effect, prolonging the duration that the antigen is exposed to immune cells [17,18]. NPs also possess immunomodulatory properties [19]. Polystyrene NPs, poly(lactic-co-glycolic acid) (PLGA), carbon nanotubes (CNTs), aluminum oxyhydroxide NPs, silicon dioxide (SiO_2_) NPs, carbon black NPs, and titanium dioxide (TiO_2_) NPs, for example, have been shown to stimulate the nucleotide-binding domain-like receptor protein 3 (NLRP3)-associated inflammasome [20]. In fact, these NPs trigger the production of reactive oxygen species (ROS) and lysosomal destabilization after they are ingested by APCs, which leads to the expulsion of phagosome contents, notably cathepsin B, a cysteine protease. NLRP3 detects this protease, which then triggers the development of the inflammasome complex [21,22,23,24,25]. Interleukins are then generated as a result of downstream signaling processes, contributing to immune cell upregulation [21,26,27,28,29,30,31]. These characteristics suggest that NPs might be useful antigen transporters and immune cell stimulators in vaccines.

Presently, a wide range of NPs, particularly liposomes, polymeric and inorganic NPs, and self-assembled protein NPs and virus-like particles (VLPs), are being explored as antigen carriers. Due to the nanosized nature of many biological systems, such as proteins and viruses, these materials provide substantial benefits [32,33]. NPs can be injected subcutaneously or intramuscularly or given through mucosal sites (oral and intranasally), penetrating capillaries and mucosal surfaces [34,35].

## 2. Nanoparticles and Nanovaccine Strategy

**Figure 1:**
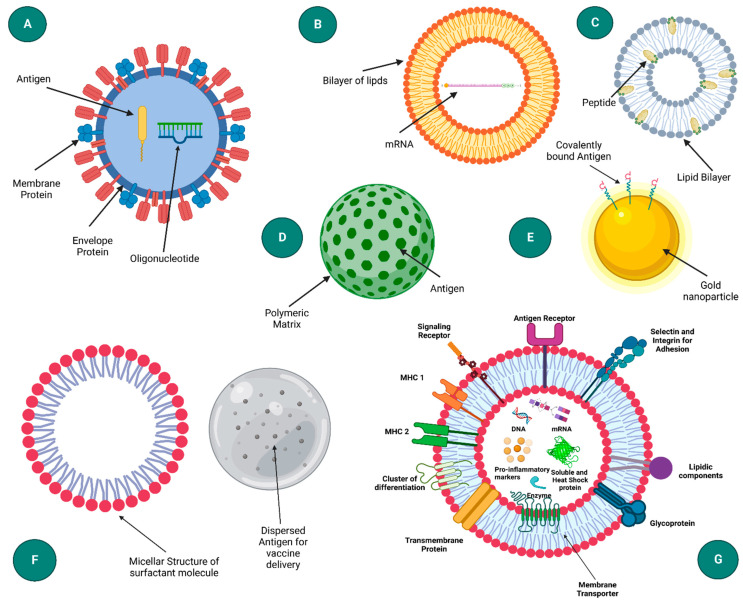
Schematic representation of different nanoparticle based delivery system: (**A**) virus-like particle, (**B**) liposome, (**C**) ISCOM (Immune stimulating complexes), (**D**) polymeric nanoparticle, (**E**) onorganic nanoparticle, (**F**) emulsion, and (**G**) exosome. (Created with Biorender.com (accessed on 28 September 2022)).

The ability of NPs to modulate immune responses to achieve desired results is essential for the formulation of vaccines using nanoparticles. To induce and heighten protective immunity, NPs can function as both a delivery system and an immune-stimulating adjuvant [36,37]. Furthermore, some important advantages of nanovaccines (vaccines based on nanoparticles (NPs) as carriers and/or adjuvants [38]) over conventional vaccines are decreasing the rate of antigenic degradation, enhancing the stability of antigens, improving the therapeutic efficacy and immunogenicity of vaccines, facilitating phagocytosis and fast processing by antigen-presenting cells (APCs), and enhancing cellular membrane penetrability [39]. It was observed that nanocarriers such as liposomes, dendrimers, and virosomes possess cytokine induction and antibody response-enhancing properties, due to which strategies have been made to deliver vaccines through nanocarriers [40]. These nanocarriers are a variety of nanomaterials with unique architectures that can be used as drug delivery systems. In addition, they improve bioavailability, stabilize and protect more sensitive agents (e.g., proteins), minimize side effects, and allow for active targeting [41]. Peptide drug conjugation will also be a better alternative to present practices, one worthy of exploration [42]. Table 1 represents the advantages and disadvantages of various nanoparticles in vaccine delivery, and Table 2 gives an overview of viral therapeutics using nanocarrier technology. Some common vaccine carriers (Figure 1) are discussed below.

### 2.1. Virus-like Particles (VLPs) and Virosomes

VLP-based vaccinations are a synthetic, biodegradable NV strategy. The particle size varies in dimension from 80 to 150 nm and possesses an empty core that can be utilized to carry drugs or antigens for targeted delivery whose membrane is made up of viral phospholipids and glycoproteins. These vaccinations exhibit a virion’s fusogenic features, which enable them to convey the trapped or cross-linked antigen to both MHC class II (CD4+) and MHC class I (CD8+) antigen-presenting pathways and APCs through receptor-mediated endocytosis [58,59,60]. VLPs are attracting significant interest due to the simplicity of their manufacture and their capacity to activate powerful immunological responses [61]. RNA of the virus and genetic code responsible for integrase are removed to abolish its virulence, so that it does not show any ill effects on host cells [62]. It is possible to produce VLPs that offer defenses against heterologous antigens in addition to the virus of origin. Pharmacologically attaching nonprotein antigens such as polysaccharides or small chemical molecules to the viral membrane can also produce bioconjugate VLPs [63].

The baculovirus expression system is the one most frequently employed for the creation of VLPs and has an excellent safety profile, since baculoviruses do not naturally infect humans. Although baculovirus expression has several advantages, it has two serious drawbacks. First, the baculovirus expression system lacks a definite ability to generate genuine hybrid mammalian glycoproteins, since insect and mammalian cell lines differ in their post-translational alteration patterns, but this limitation can be surpassed by generating a “humanized” insect cell line that can further generate mammalian cell lines, such as 4-galactosyltransferase, β1 and α-2,6-sialtransferase, to express terminally sialylated and galactosylated glycoproteins [64,65]. Second, spontaneous cell death and insect cell lysis occur after infection with baculovirus, which can lead to problems in the secretion of required proteins, and the chances of degradation increase abruptly. In an effort to solve this issue, random mutations were used to create a nonlytic baculovirus, which reduced expressed protein degradation and also reduced cell lysis by a factor of almost ten [15].

A number of vaccines that use VLPs have also been approved for use against the hepatitis B virus (HBV) and HPV. The recombinant HBV vaccine is a huge achievement since it is the first commercially available vaccine based on VLPs. The vaccine contains a recombinant hepatitis B virus core antigen (HBsAg), and for the production of immunogenic VLPs, yeast (*Pichia pastoris*, *Saccharomyces cerevisiae* and *Hansenula polymorpha*) and mammalian cells (Chinese hamster ovary cell line [CHO]) were used as expression hosts [66,67,68,69,70]. The VLP structure and production process are both affected by the choice of the host cell. GenHevac B^®^ by Pasteur- Mérieux Aventis and Sci-B-Vac™ by SciGenin Israel are examples of recombinant HBsAg based on VLP [71].

Out of all types of papillomaviruses known to infect humans, approximately 15 are known to cause cervical cancer, and approximately 70% of cases worldwide are caused by the 16 and 18 HPV types [72]. For this reason, VLP-based HPV vaccines that have been approved contain VLPs of HPV-16 and HPV-18. The first VLP-based HPV vaccine to be approved by the FDA was Gardasil in 2006. The Gardasil vaccine is a quadrivalent (HPV types 6, 11, 16, and 18) VLP-based vaccine derived from recombinant L1 produced in *S. cerevisiae* [73]. Addressing the ongoing COVID-19 pandemic, researchers have found that VLP-mediated SARS-CoV-2 vaccines are excessively immunogenic and possess properties that directly activate B-cells for the production of neutralizing antibodies in mice. The concentrations of coronavirus-neutralizing antibodies were also shown to be dramatically enhanced by VLPs encapsulated with spike proteins of SARS-CoV and MERS-CoV in preclinical investigations. Similarly, in the case of SARS-CoV-2, the spike (S) protein is the prime candidate for vaccine formulation and neutralizing antibodies and one of the essential elements in virus-host cell fusion and receptor binding [74,75]. The COVID-19 VLP (Serum Institute of India) and GBP510 (SK Bioscience, CEPI) vaccines are two VLP-based vaccines under phase I/II clinical trials [76]. Another virulent marker that may help designate pharmacotherapy approaches is the SARS-CoV-2 envelope (E) protein. A protein called haem agglutininesterase (HE) is also expressed in certain SARS-CoV-2 [77]. The lectin motif of HE protein promotes virus-host cell adhesion. To synthesize virosome vaccines, lipid membranes and particular membrane antigens of SARS-CoV-2 were isolated. Virosome NPs are a potential delivery system for SARS-CoV-2 vaccination [78]. The European MI Matrix Company is operating on the Transvac 2 project, a virosomal-based vaccine [79]. Virosomes have properties of activating both helper T cells and cytotoxic T lymphocytes against vaccine candidates, which makes them very prominent nanocarriers for vaccine delivery. The concept of immune-potentiating reconstituted influenza virosomes (IRIVs) has been extensively used in the production of vaccines against hepatitis A and influenza virus. Such IRIV can also be used in the treatment of certain cancers and autoimmune and allergic disorders [80,81]. The virosome-mediated vaccine delivery system has also been used in the treatment of other deadly diseases, such as Ebola, hemorrhagic fever, visceral leishmaniasis, Lassa hemorrhagic fever, and HIV [82].

### 2.2. Liposomes

Liposomes are membranous vesicles that are spherical, single or multilayered with dimensions of approximately 50–500 nm [83,84]. They are composed of amphipathic phospholipid molecules that self-assemble around a hydrophilic core that is hollow and filled with liquid within the lipid bilayer, similar to VLPs. Because of this, liposomes can accommodate either hydrophilic or hydrophobic molecules within their phospholipid bilayers [44,85,86,87]. Antigen and adjuvant can be delivered to the same APC simultaneously via liposomes, which is one of the main advantages of liposomes. Furthermore, liposomes may protect antigens against degradation, enhance their uptake by APCs, promote their release into the cytosol and even stimulate the immune system [44,87]. Additionally, liposomes can be divided into cationic, anionic, and neutral types, with cationic liposomes being the most efficient when compared to others. Catalytic liposomes have been praised, in particular, for superior cellular uptake, antigen transport, and activating DCs and macrophages among cationic, anionic, and neutral liposomes [88,89,90].

According to their chemical makeup, liposomes can carry DNA, RNA, peptides, proteins, haptens, and carbohydrates by encapsulating them in the core of the aqueous layer, whereas intercalation or surface-attaching mechanisms are applied for the targeted delivery of water-insoluble substances such as hydrophobic drugs, additional lipids, dyes and antibodies. In particular, antigen or medication protection against pH fluctuations and extracellular breakdown is provided by liposomal entrapment [91,92].

Nevertheless, the intrinsic adjuvant characteristics of liposomes have been validated by Gregoriadis et al. [93]. Mai Y et al. from their investigation showed that, in experiments, vaccinated rodents exhibited robust antibody immunological responsiveness to antigens such as diphtheria toxoid. In addition, it was shown that mice administered liposome-based vaccinations did not experience the adverse reactions caused by traditional vaccine additives, such as nodule formation [94]. The ratio of CD4+ and CD8a+ T cells in the LPC/mRNA group increased. Th1 cells usually secrete cytokines involved in the innate cellular immune response, such as IL-2, to promote the activation and proliferation of T cells. Mice with the L-TriADJ (polyphosphazene) combination encapsulated in cationic liposomes were intranasally administered by Ellen K. Wasan et al., which culminated in heightened immunoregulatory activation [95]. Exposure of RAW267.4 mouse macrophage cells to TriAdj alone vs. L-TriAdj indicated that DDAB/DOPE (50:50) and DDAB/EPC/cholesterol (40:50:10) complexation reduced TriAdj toxicity. Stable particles (<200 nm over 24 h) showed mucin binding of DDAB/DOPE + TriAdj was greater than DDAB/EPC/DOPE + TriAdj. The balance of charged polyelectrolyte components incorporated into the lipidic adjuvant promoted self-assembly and condensation, and an overall cationic charge inhibited gross aggregation and facilitated mucin interaction, as indicated by its effects on the measured zeta potentials. L-TriAdj significantly increased the immune response of mice administered adjuvant compared with TriAdj alone, with a dose-response proportional to triple adjuvant content and an overall balanced Th1/Th2 immune response reflecting both systemic and mucosal immunity [95]. In another study by Espinosa et al., a novel cationic liposomal adjuvant system, CAF09, combined with a full-length recombinant Plasmodium falciparum circumsporozoite (Pf rCSP) protein, was studied to determine its immunogenicity and protective capacity by using newly developed transgenic rodent malaria parasites. This liposome-based protein-in-adjuvant formulation was capable of inducing strong antibodies and CD8+ T cells that are capable of suppressing parasite infection and liver stages, thereby conferring durable sterilization [96].

Liposome NPs that have been commercially approved for human use are limited to virosomal vaccines, Inflexal V and Epaxal, for use against hepatitis A and influenza, and flu vaccines, such as Invivac and NasalFlu [36]. Liposomes co-formulated with immunomodulators can serve as effective adjuvants in addition to delivering inactivated viral vaccines (such as Infexal VTM and EpaxalTM) [81]. For example, RTS, S/As01 (for malaria) and Shingrix (for Singles) are the only FDA-approved vaccines where the As01 liposomal adjuvants system contains MPLA (monophosphoryl lipid A) and QS-21 (saponin from Quillaja saponaria) [89]. The creation of receptor-targeted nanocarriers in tumor immunology that can specifically kill cancer cells without off-target effects has been the focus of recent studies [82]. Even lyotropic liquid crystals can be a suitable option for mRNA delivery [97]. Also, intranasal delivery of vaccines will provide a better option for vaccine delivery in respiratory infections [98,99].

### 2.3. Immunostimulatory Complexes (ISCOM)

Immune-stimulatory complexes (ISCOMs) are other vaccine delivery vehicles that have been shown to have strong adjuvant properties in clinical trials. These micelles (40 nm in size) contain colloidal saponin (Quil A or its purified components, most commonly saponin) [100], cholesterol, and phospholipids (phosphatidylcholine or phosphatidylethanolamine) and are considered self-adjuvanted vaccine delivery systems [101]. A classic use of ISCOMs has been to entrap viral envelope proteins, including those from hepatitis B, influenza viruses, and HSV-1 [102,103]. Similarly, another vaccine delivery vehicle that is similar to ISCOMs in adjuvants is also available, but in this type, a viral protein is not available. However, when an antigen is added in later stages, hydrophilic antigens can be entrapped. Such a complex is known as empty ISCOMs or ISCOMATRIX™ [104]. ISCOMs have a unique structure due to the interaction between saponins and cholesterol. In the ISCOMs and ISCOM matrix, cholesterol is a crucial component, as is phospholipids, typically phosphatidylethanolamine [105] or egg-derived phosphatidylcholine [106]. According to some studies, phospholipids provide a looser fitting ISCOM than cholesterol alone, allowing amphipathic molecules, such as viral membrane proteins, to be inserted [106]. ISCOMs and ISCOMATRIX™ vaccines are made using diverse antigens, including those derived from Newcastle disease, HIV, influenza, and HPV [107,108,109]. The preparation of ISCOMs normally involves detergent removal of mixed micelles that consist of Quil A, lipids, the antigen, and an appropriate detergent. Generally, ISCOMs are composed of 60–70% Quil A, 10–15% lipids, and 5–20% protein. Depending on how high the concentration of detergent is, dialysis or ultracentrifugation can be used to remove the detergent [110]. An alternative method for preparing ISCOM-matrix was recently described by using Quil A solution to dissolve lipid films [110].

Cibulski et al. evaluated the immunological activities of ISCOMs formulated using cholesterol, phospholipids, and OVA as a model antigen derived from Quillaja brasiliensis (QB-90). When IQB-90 was subcutaneously administered, specific IgG1 and IgG2 antibodies were produced, as well as T-cell proliferation and increased Th1 cytokine production. An advantage over traditional ISCOMs based on Quil A is that intranasal delivery stimulates serum IgG, IgG1, and mucosal IgA responses at distal systemic sites [111].

### 2.4. Polymeric Nanoparticles

Polymeric NPs are remarkably fascinating for the administration of vaccines due to their biodegradability, stability, safety, ease, surface modification, biocompatibility and predictability [112,113,114]. The polymer-based delivery system offers some advantages, including delayed release, resistance to environmental degradation and enzymatic degradation and adjuvant effects. The two main categories of polymeric NPs are natural polymeric NPs and synthetic NPs [115,116]. In natural nanoparticles, hyaluronic acid, chitosan and alginate are the most commonly used, and the latter’s conjugation of animal venom toxins with its NPs is currently the main focus of research [56,117,118,119,120]. DNA-encoded vaccines and vaccines against hepatitis B virus (HBV) may also be prepared by using them. These natural NPs can also provide better cellular interactions, targeted therapy and a more effective drug delivery interface [121]. Furthermore, since synthetic polymeric NPs have a slow biodegradation rate, they can accomplish some goals, such as entrapping antigens for delivery or sustaining antigen release. A variety of polymers may be used to prepare these polymeric NPs, such as polysaccharides, poly (amino acids), and poly (α-hydroxy acids), to produce vesicles that can either encapsulate or exhibit antigens [122,123,124]. Poly(α-hydroxy acids), such as poly(d, l-lactic-co glycolic acid) (PLGA) and poly(d, l-lactide-co-glycolide) (PLG), are commonly used [122,123,124], with PLGA being extensively used due to its significant properties, such as sustained release, protection of drugs or antigens from degradation and the ability to co-encapsulate immune-potentiators and antigens, which have attracted much attention [125]. These NPs are often synthesized using a double emulsion-solvent evaporation technique. By dissolving the polymer in an organic solvent (methylene chloride or ethyl acetate), adding the antigen followed by vortexing will result in a primary emulsion. This emulsion is then converted to a water-in-oil-in-water type emulsion by the addition of an emulsifying agent (e.g., polyvinyl pyrrolidine or polyvinyl alcohol), resulting in precipitation of the polymer around the antigen. Furthermore, to prevent degradation of the polymer due to water-catalyzed ester hydrolysis, the solution is left to evaporate the solvent and finally dried. The main limitation of this method is the low efficiency of the entrapment of the antigen, which may result in protein denaturation at the oil-water interface. To correct this, a surfactant or sugars (sucrose and trehalose) are added as a stabilizer, which can hydrate the protein, preventing denaturation [15]. Nanoprecipitation is another extensively used method for the preparation of synthetic polymer nanoparticle-based vaccine systems. Here, a polymer and a drug should ideally dissolve in the first system (the solvent) but not in the second system (the nonsolvent). Adding a polymer solution to a nonsolvent results in nanoprecipitation by rapid desolvation. When the polymer-containing solvent diffuses into the dispersing medium, it precipitates, entrapping the drug immediately [126].

Another important polymer in polymeric NPs is polyethyleneimine (PEI), which is known to be a cationic polymer and is extensively used in DNA delivery and gene therapy applications. Apart from poly-L-lysine (PLL), PEI is an acceptable nonviral nucleic acid transfer agent [127]. A PEI-based vaccine could be made by several different methods: directly binding with antigens, coating on antigen-loaded NPs/MPs, coating existing particles with antigens absorbed on their surface, or encapsulating preexisting particles with antigens. As part of these processes, cytokines and ligands could also be added to vaccines based on desired applications. Recent studies have also found that vaccines containing PEI as the immunostimulant are effective at treating infections and tumors [128]. In one study by Bivas-Benita et al., a DNA vaccine encoded with Mtb latency antigen Rv1733c was evaluated in this study to assess its immunogenicity, as well as its effects on host immunity when delivered or co-formulated with poly(d,l-lactide-co-glycolide) (PLGA)–polyethyleneimine (PEI) nanoparticles (NPs). In this study, researchers vaccinated BALB/c female mice three times at three-week intervals. The mice were immunized intramuscularly or by endotracheal aerosol application. Three weeks after the last vaccination, boosting was performed by administering Rv1733c protein intramuscularly in incomplete Freund’s adjuvant (IFA) [129,130]. Control animals were administered the Rv1733c protein subcutaneously in IFA using the same vaccination regimen. In comparison with lipopolysaccharide (LPS) stimulation, the NP matured human dendritic cells and stimulated their secretion of IL-12 and TNF-α. T-cell proliferation and IFN-γ secretion were enhanced in mice by Rv1733c DNA prime and Rv1733c protein boost in response to Rv1733c and *Mtb* hypoxic lysate. Adsorption of Rv1733c DNA on PLGA-PEI nanoparticles and application to the lungs increased T-cell proliferation and IFN-γ production more than intramuscular vaccinations. Pulmonary priming with np-adsorbed Rv1733 cDNA followed by Rv1733c protein boosted the greatest immunogenicity. According to these findings, PLGA–PEI np can be used to deliver DNA vaccines through pulmonary delivery to enhance T-cell responses in a DNA prime/protein boost vaccine regimen [129].

### 2.5. Inorganic Nanoparticles

Studies have been conducted on inorganic nanoparticles for their potential use in vaccines. Inorganic NPs have benefits for vaccine distribution due to their hard structure and predictable fabrication; however, they are predominantly nonbiodegradable. To enhance the immune response, inorganic NPs have been used as adjuvants and delivery vehicles. The main inorganic NPs are carbon, silica, calcium phosphate, aluminum-based, gold and magnetic nanoparticles. Several studies have been performed on how the size and shape of an inorganic nanoparticle can affect vaccine delivery [131]. Tsai et al. reported that macrophage expression levels of cytokines were affected by nanoparticle diameter after incubation with gold and silver nanoparticles [132]. Plebanski et al. found that antigen-immobilized polystyrene beads affected the balance of type 1/type 2 cytokines [40]. Based on these data, nanoparticles of suitable size might be useful as adjuvants beyond their use as antigen carriers. In contrast to the size effect, there are very few studies looking at the effect of shape on immunological response. Maysinger et al. reported that the inflammatory response in microglial cells is shape-dependent [133]. The production of the inflammatory cytokine interleukin-1 was shown to be elevated by nanourchins but not by spherical or rod-shaped particles. Based on these differences in cytokine production in response to nanoparticles of different shapes, it appears that nanoparticle shape may affect the immune response in a desirable manner.

Gold nanoparticles (AuNPs) are easily fabricated into a variety of shapes (spherical, rods, cubic, etc.). These types of nanoparticles can induce humoral and cellular responses [131]. By conjugating the antigen to the surface of gold nanorods, respiratory syncytial virus antigens can be delivered [134]. Other varieties of gold nanoparticles have been utilized as DNA vaccine adjuvants for human immunodeficiency virus (HIV) or as carriers for antigens derived from other viruses, such as influenza [54] and foot-and-mouth disease [135]. Carbon nanoparticles can be synthesized into nanotubes and mesoporous spheres [136,137,138,139], and to enhance the IgG response, multiple copies of protein and peptide antigens can be conjugated to carbon nanoparticles (CNTs) [137]. Silica-based nanoparticles are another promising nanocarrier in vaccine delivery due to their ability to target a selective tumor [140,141] and real-time multimodal imaging [142]. These types of nanoparticles are prepared by adjusting their structural parameters to selectively alter their interaction with cells [143]. Another type of inorganic nanoparticle is calcium phosphate nanoparticles, which are formed when calcium chloride, dibasic sodium phosphate, and sodium citrate are mixed under certain conditions [144,145]. They are nontoxic and can be formed into a size between 50 and 100 nm [146]. The nanoparticles show excellent biocompatibility and are useful as adjuvants for DNA vaccines and mucosal immunity [144,145,146,147].

Based on a study by Xu et al., gold nanorods were modified with poly(diallydimethylammoniumchloride) or polyethyleneimine, which significantly enhanced cellular and humoral immunity, as well as T-cell proliferation, by activating antigen-presenting cells, compared with naked HIV envelope plasmid DNA treatment in vivo [148]. Wang and his team conjugated trimetric influenza hemagglutinin on gold nanoparticles and used the TLR-5 agonist flagellin as a particulate adjuvant. Antigen-specific proliferation of CD4+ cells and CD8+ cells was activated following intranasal vaccination in mice, leading to an increase in influenza-specific IgA and IgG levels [149]. Several studies have also found that different types of inorganic nanoparticles can cause toxic effects on the reproductive system of male rodents [150].

### 2.6. Emulsion

For decades, emulsions have been extensively used in the formulation of vaccines, and currently, they are being researched for use in vaccine delivery systems. Since emulsions are thermodynamically unstable, they can separate into two different phases, i.e., oil and water [151,152,153,154]. They can be used to give vaccinations by integrating antigens into their structure or by blending with the antigens. Compared to larger-sized emulsions, nanoemulsions perform better because they can effectively deliver the antigen to the APCs due to their ability to penetrate the nasal mucosa. Most of these NPs are employed as adjuvants in the preparation of vaccines [155]. A popular oil-in-water emulsion with approval, MF59TM, is commonly utilized in the formulation of vaccines. This vaccine adjuvant has undergone extensive research for use in influenza vaccinations and is both safe and effective [36,155].

Furthermore, microemulsions (MEs) are newly developed vaccine delivery systems that possess higher target specificity and more therapeutic effectiveness than nanoemulsions in terms of spontaneous production and thermodynamic stability [156,157,158]. Researchers have found that the immune-enhancing properties of flavonoid compounds and intranasal adjuvants for influenza vaccines can be improved by preparing ME. Similarly, no topical reactions were triggered by the ME formulation of propylene glycol, polysorbate 80, and isopropyl myristate as adjuvants for bluetongue and rabies virus vaccinations [159,160]. The ME preparation was the best alternative for rabies vaccination while exhibiting a poor antibody response for the bluetongue vaccine because the adjuvanticity of the mechanisms was anticipated to be controlled by the mean particle size. Maximum cellular absorption was achieved by particles with a size of 20–50 nm, which were also more easily absorbed into the lymphatic system and more effectively activated the dendritic cells that are found in the lymph nodes [161,162]. Emulsifiers such as Solutol HS15 (Macrogol 15 hydroxystearate) and Cremophor (CreEL, Polyoxyl 35 castor oil) act as crucial components for the spontaneous preparation of effective MEs, which basically reduce the interfacial tension and impart stability to the emulsion [163,164].

To protect against Acinetobacter baumannii infections, Yang et al. designed a vaccine combining the OmpK/Omp22 fusion protein with MF59, which is an oil-in-water (o/w) emulsion containing squalene (4.3% *v*/*v*) and two surfactants, polysorbate 80 (0.5% *v*/*v*, Tween 80) and sorbitan trioleate (0.5% *v*/*v*, Span 85), which are emulsified in citrate buffer to produce droplets of ~160 nanometers in size. BALB/c mice were immunized intratracheally along with two booster doses, resulting in antigen-specific antibodies, lower bacterial loads in the blood and lung tissues, and a reduction in inflammatory cytokines [165]. This study shows that MF59 is an effective adjuvant.

### 2.7. Nanogels

Nanogels or hydrogel NPs are also used as vaccine delivery vehicles and are known to have a particle size ranging from 1 to 1000 nm and swelling networks made of amphiphilic or hydrophilic polyionic polymers, which may be obtained naturally or artificially. The delivery of vaccines has become very beneficial due to their nanoscale size, which enhances the bioavailability of biological substances by facilitating their movement through biological barriers. This lengthens their action in the target region [166]. The distribution of vaccines benefits from the nanoscale size, which enhances the bioavailability of biological substances by facilitating their movement through biological barriers. This lengthens their action in the target region [167]. The particle size may also have an impact on the transport across mucosal surfaces. An appealing and difficult route for immunization is through the mucosae [168]. Surface charge may have an impact on a vaccine formulation’s bioadhesivity, entrapment effectiveness, % loading, stability, and in vivo immunogenic efficacy. Hydrophobicity and hydrophilicity are also maintained for the maximum targeted delivery of vaccines [169]. Nanogels are specifically made to overcome numerous barriers and reach the circulation intact depending on the method of administration. Opsonization of the nanogels, which is followed by their clearance through MPS organs such as the liver and spleen, where they are picked up by local monocytes and macrophages, is one of the biggest barriers to establishing extended circulation [170]. The most commonly used biopolymers for nanogel preparation are dextran, mannan, pullulan, alginate, dextrin, chitosan, hyaluronic acid, poly-L-lysine, and heparin, whereas synthetic polymers with biodegradability and biocompatibility are poly-D,L-lactic acid (PLA), poly(ε-caprolactone) (PCL), polyglycolic acid (PGA), polymethyl acrylate (PMMA), and poly-D,L-lactic-co-glycolic, which are approved by Food Drug and Administration for efficient vaccine delivery [171,172,173]. Gao et al. developed highly efficient tumor-targeted drug delivery mesenchymal stem cell membrane-coated gelatin nanogels (SCMGs) derived from bone marrow. SCMGs show an excellent cell mimicking cancer targeting capacity in vitro. They then loaded hydrochloride doxorubicin (DOX), an anticancer drug, into the gelatin nanogels. When injected intravenously, SCMG targeted and accumulated in the tumor tissues. SCMGs-DOX displayed significantly greater antitumor therapeutic efficiency than gelatin-DOX or free-DOX [174].

### 2.8. Lipid Nanoparticles

Several nanoparticle systems derived from biocompatible polymers, lipids and oils have been developed to improve the bioavailability of drugs by enhancing the permeability of the drug or overcoming the first-pass effect [175]. Among these particles, lipid-based nanoparticles pose a minimum threat for in vivo applications, and lipid-based nanoassemblies have been used to deliver DNA and RNA as well as drugs [176,177,178]. This has made lipid nanoparticles the most promising carriers for oral drug delivery [179]. Not only can it enhance permeability, but it also influences the absorption of a drug by preventing drug precipitation on intestinal dilution, increasing solubilization capacity, reducing CYP enzymes, inhibiting efflux transporters and enhancing chylomicron production and lymphatic transport [180,181,182]. The preparation of lipid nanoparticles (LNs) is performed by using low chronic and acute toxicity physiological lipids (biodegradable and biocompatible, which is similar to liposomes and nanoemulsions). In addition, their solid matrix offers the same protection as polymeric nanoparticles against chemical degradation under harsh biological environments and provides the maximum flexibility in regard to modifying drug release profiles [183]. However, in polymeric nanoparticles, toxic effects may be caused by the in vivo degradation of the polymer [184]. There are two types of LNs with a solid matrix, viz., solid lipid nanoparticles (SLNs), also known as first-generation lipid nanoparticles, and nanostructured lipid carriers (NLCs), or second-generation SLNs [176,179,185]. Solid lipid nanoparticles consist of solid lipid cores that range between 50 and 1000 nm in diameter (measured with photon correlation spectroscopy, PCS) [186]. They are stabilized by suitable surfactants, and these lipidic materials may contain complex glyceride mixtures, purified triglycerides, or waxes that are solid at room temperature and human body temperature [184]. Here, the drug is mainly dispersed in molecular form, for example, between the fatty acid chains of the glycerides. Moreover, SLNs serve as an alternative carrier system to other carriers, such as emulsions and liposomes [187]. However, SLNs also have a few disadvantages, such as poor loading capacity, relatively high water content (70–99.9%) and drug expulsion during storage after polymorphic transitions [187,188,189,190]. NLCs were then introduced to overcome the limitations present in SLNs. In NLCs, both solids and liquid lipids are used, and since their structures are different, they are unable to form a perfect crystal. A matrix with this arrangement has many imperfections, which allows more drugs in molecular form and in amorphous clusters to be accommodated [191,192,193]. The solid matrix of NLCs reduces particle coalescence and allows drugs to be more strongly immobilized than in emulsions [194]. Lipid nanoparticles are also effective delivery systems for small molecules, mRNA and siRNA. Notably, during the recent SARS-CoV-2 pandemic, two lipid nanoparticle-based mRNA vaccines were clinically tested and approved for emergency use in late 2020, viz., the Moderna COVID-19 (mRNA-1273) vaccine and BNT162b from Pfizer-BioNTech. These vaccines are nonviral mRNA-lipid nanoscale complex vaccines encoding some form of the SARS-CoV-2 spike protein (Moderna) or its receptor binding domain (Pfizer/BioNtech) [49,195]. Preclinical and clinical research of lipid nanoparticle–mRNA formulations has led to the rapid development of COVID-19 lipid nanoparticle–mRNA vaccines. Within a month of the SARS-CoV-2 genome sequence being available, the clinical-grade COVID-19 vaccine mRNA-1273 was developed. Phases I, II, and III of human trials were initiated approximately 2, 5, and 6 months after sequence availability. The FDA granted mRNA-1273 an Emergency Use Authorization within one year, and the EMA granted it conditional marketing approval [196,197].

## 3. Characterization of Nanoparticles

A multidisciplinary approach is required to characterize these nanomedicines because of their inherent complexity. Nanovaccines (NV) can be accurately characterized through physicochemical, immunological, and toxicological tests. In NVs, the main objective is to facilitate an efficient immune response to the cargo while reducing nanocarrier (NC) reactions. Each critical parameter must be evaluated according to its mechanism of action and the impact it has on the performance of every component in the formulation. Additionally, mRNA- and protein-based NVs will require different characterization techniques. Moreover, the immunogenicity and safety of the formulation are influenced by its physicochemical properties [198,199]. To prevent variation between (or within) batches, NPs used as vaccine delivery vehicles are characterized after being synthesized concerning their structure, size and content of the nanoparticle formulations. The variation could be a result of a polydisperse population of NPs, inadequate particle development, the deposition of harmful substances, or contamination. Several techniques are used to assess uniformity in colloidal solutions to maintain a homogeneous population. Because NPs are spherical in shape, spatial homogeneity throughout the NP is crucial, since the spherical volume could alter the proportion of antigen that is conjugated or internalized onto the surface, as well as the vaccine’s immunizing dose. Therefore, several techniques, such as transmission electron microscopy (TEM), scanning electron microscopy (SEM) and atomic force microscopy (AFM), are used to determine the size and structure of particles [102,200,201], and the size distribution of small particles such as NPs is evaluated by dynamic light scattering (DLS) [201]. Next, the concentration of antigen present is measured using the following methods: dot blotting, Lowry and Bradford assays, sodium dodecyl sulfate-polyacrylamide gel electrophoresis, density gradient centrifugation, enzyme-linked immunosorbent assays or Western blotting [202,203,204,205,206]. Furthermore, if any of the reagents in the NP are toxic in large doses, it is desirable to evaluate the composition of the NP. This is notably correct with Quil A, a vital ISCOM component that, at high enough quantities, can have a hemolytic impact and that can be detected using reversed-phase high-performance liquid chromatography or a rocket electrophoresis assay [200,207]. A phosphorus assay and gas chromatography are used to evaluate other ISCOM components, such as cholesterol and phospholipids, respectively [200]. A variety of metals (and nonmetals), including gold, can be incorporated as NPs and can be assessed via inductively coupled plasma-mass spectrometry or instrumental neutron activation analysis [208,209]. The methods used to characterize NPs are compiled in Table 3.

## 4. Exosome-Based Vaccine Delivery System

Extracellular vesicles (EVs) are a class of bi-layered membrane vesicles with a diameter of less than one nanometer that are created by practically all cells. Body fluids such as blood, saliva and breast milk naturally contain EVs. Traditionally, EVs are categorized according to their size, chemical content, and biogenesis route. According to their biosynthesis, exosomes and microvesicles are the two primary categories of EVs that may be widely defined. Multivesicular bodies (MVBs) produce extracellular vesicles (EVs) such as exosomes, which are 30–150 nm in diameter before being expelled upon MVB integration with the plasma membrane. The outward budding of the plasma membrane, which is controlled by the movement of phospholipids, results in the formation of microvesicles [231]. Exosomes have the ability to fuse with recipient cell plasma membranes in the extracellular region, releasing their bundled substance into the cytosol. Exosomes are remarkably varied transporters of a broad range of molecules, including nucleic acids such as miRNAs and mRNAs, proteins and lipids. The trafficking of these molecules might take place within the exosome itself or by adhesion to the surface of recipient molecules, as shown by the example of major histocompatibility complex (MHC) molecules [232]. Exosomes are released by healthy cells under biologically appropriate conditions and are involved in a variety of biological processes, including cell signaling, by fostering the transport and distribution of numerous particles that can potentiate significant mechanisms such as stress response, differentiation, and growth [233,234].

It is possible that exosome-based vaccines will be widely used therapies in the future because of their involvement in disease development, their function in preventing viral infections, and their ability to induce a host immune response. Exosomes and viruses are comparable in terms of size, molecular make-up, biomolecule transfer methods, ability to enter host cells, biosynthesis and growth of viruses in host cells. One well-known example is human immunodeficiency virus (HIV-1), which accelerates its spread throughout the host’s body by manipulating EV biogenesis via the ESCRT pathway. As a result of changes in EV cargo during viral infection, including the transfer of viral particles into uninfected cells and modulation of immune response, researchers have also characterized EVs, investigated their therapeutic potential (as a drug delivery system) or used them to present antigens for safe vaccine design [235]. Additionally, EVs are excellent vaccine candidates due to their stability, vascular permeability, biodistribution, and solubility. A safe vaccine must be created using the right strategy. As part of several in vivo studies, EVs have been assessed for immunogenicity and toxicity [236,237]. BALB/c mice did not show signs of hepatotoxicity or inflammation induction by EVs made from human embryonic kidney Expi293F cells, whereas EVs made from CD81+/CD9+/CD63+ cells showed no effect on mRNA expression levels in HepG2 cells [237]. An in vivo study confirmed the safety of EVs by showing that CD63+/TSG101+ EVs from 293T cells of human embryonic kidneys did not cause toxicity or immunological response. The main characteristics of EV-based vaccines, such as their capacity to cause low immunogenicity, indicate that EVs can be employed in the production of vaccines in a secure and effective manner. Comparing EVs to alternative delivery agents such as viral vectors or lipid-based NPs (LNPs), EVs have a benefit, i.e., in maintaining naive antigen structure and gaining access to all organs through physiological fluids. Therefore, because they have a highly effective antigen-presenting system and excellent biosafety, modified EVs meet the requirements for effective vaccination [235,238,239]. Exosomes that are used as food are often extracted from milk and vegetables. More research is required to determine their toxicity. Despite their outstanding stability under a variety of circumstances, they can be used for large-scale synthesis and effective loading of hydrophilic and hydrophobic compounds. Exosomes can be given orally, intravenously, intraperitoneally, subcutaneously, and intranasally. Although intravenous injection is the most common method for delivering drug-loaded exosomes to target cells, this method hinders the drug’s ability to accumulate in tumor tissues because the exosomes are quickly removed from the circulation and are instead deposited in the spleen and liver [240]. Exosomes that have been loaded with medication can also be administered locally intravenously and topically. Drugs can be administered via an intranasal route to the central nervous system, whereas exosomes made from milk or plant extracts can be taken orally [241]. Exosomes can be loaded with therapeutic compounds using a variety of techniques, including pre- and post-loading procedures. These take place, respectively, during exosome biogenesis and following exosome isolation. The therapeutic chemicals are packaged into cells using transfection, coincubation, or genetic engineering in the preloading method and then loaded into the exosomes that the cells create utilizing cellular sorting machinery. Through physical and chemical mechanisms that cause the formation of momentary pores in the lipid bilayer to increase membrane permeability and promote the uptake of therapeutic agents, the post-loading approach involves the direct incorporation of molecules into the exosomes after their isolation. Post-loading methods include electroporation, sonication, simple incubation, extrusion, freeze-thaw cycles, click chemistry, and saponin treatment [240,242].

### 4.1. Exosome-Based Vaccine Delivery System for Viral Disease

**Figure 2:**
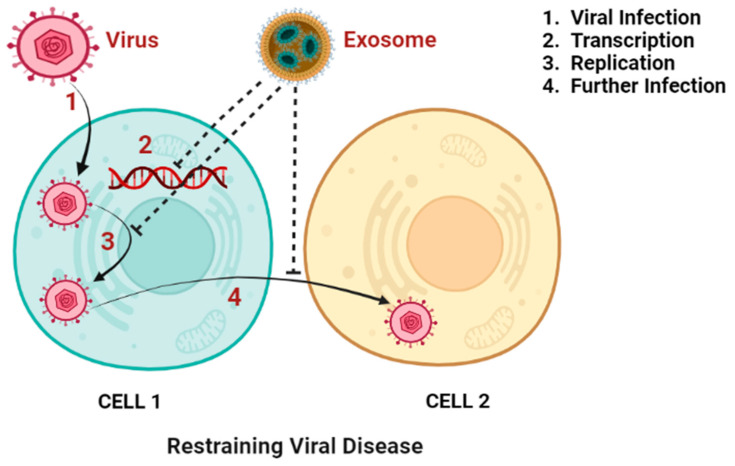
Depiction of the role of exosomes in restraining viral disease by suppressing viral infection, transcription, replication, and further infection. (Created with Biorender.com (accessed on 28 September 2022)).

Exosomes may carry and distribute substances to target cells in infectious diseases, making them a double-edged sword [242]. Exosomes induce immunological responses that provide pathogen protection while also being crucial to the pathophysiology of infection. This effect can be observed in the setting of viral infections, when exosomes produced by infected cells have the ability to both spread viral material to nearby cells and elicit an antiviral immune response. According to the “Trojan exosome” hypothesis proposed by Gould et al., which explores the evolutionary parallels between viruses and exosomes in terms of their manufacturing and transmission methods, exosomes could be exploited as a tool for an HIV vaccine [231]. Nef is an HIV protein that is involved in numerous cellular processes, including vesicular trafficking and the survival of infected cells. To develop an exosome-based vaccination, a Nef mutant (Nefmut) was introduced to exosomes. In this case, mice developed CTL immune responses to a number of viral antigens, including those for HIV, Ebola, influenza, HBV, and hepatitis C virus, after DCs absorbed Nefmut exosomes, which then presented the antigens (HCV) [243,244]. Several HBV-encoded proteins and miRNAs that control host cell gene expression are found in exosomes produced by HBV-infected cells. This clarifies the potential application of exosomes in the study of HBV transmission and host-HBV interactions [231]. Figure 2 represents how exosomes hinder viral disease.

Additionally showing promising results was a vaccination formulation that used unaltered exosomes as adjuvants for the recombinant HBV antigen, with exosomes inducing a Th1 immune response and raising mouse IFN-g levels as a result. More study is needed to identify potential therapeutic targets for HBV vaccine candidates that use exosomes as adjuvants or delivery mechanisms. Despite the wide variety of vaccine types available for influenza infection, studies have shown that exosomes can be used as a unique platform for developing influenza vaccines, with benefits above those from conventional vaccinations. For instance, exosomes released from the airways during influenza infection may contain host proteins that have anti-influenza characteristics and may stimulate immunological responses [231]. Exosomes produced by infected cells were found to have proteins that were comparable to those found in influenza virions, according to a study utilizing LC-MS/MS, suggesting a different route for the infection of fresh host cells for infecting newly formed host cells. High amounts of miR-483-3p are seen in the lungs and serum-derived exosomes of influenza-infected mice, and this is connected to the release of proinflammatory cytokines [231,245]. According to the researchers, more study is needed to determine whether miR-483-3p translocation contributes to the inflammatory pathophysiology of influenza virus infection or the activation of innate immune responses. EVs produced by gram-negative bacteria called outer membrane vesicles (OMVs) are another exosome-based vaccination approach to combat the influenza virus [245].

### 4.2. Exosome-Based Treatment for Nonviral Disease

Bacterial exosomes and outer membrane vesicles (OMVs) have been found to be powerful immune modulators instead of pathogenesis-inducing agents. As a potential venue for multiple contagious disease vaccines, it has become increasingly popular to synthesize EVs from gram-positive bacteria. Staphylococcus aureus-derived EVs have been modified to be nontoxic and serve as potential vaccine candidates. Genetically modified EVs have been shown to display immunogenic effects and protect mice against *S. aureus*-induced fatal sepsis. Additionally, when *Streptococcus pneumoniae*-derived EVs were cultured with murine DCs, they internalized quickly and increased the production of the inflammatory mediator tumor necrosis factor (TNF)-a [231]. *Schistosoma mansoni* is the primary parasite that causes the widespread disease schistosomiasis, which kills more than 280,000 people each year worldwide. There is currently no vaccine against schistosomiasis, which emphasizes the importance of developing such a vaccine. Exosome immunization against *S. mansoni* infection has only been proposed by a small number of writers [246,247]. Exosomes produced by mature *S. mansoni* worms contain miRNAs and proteins crucial in host-parasite interactions, including nutrition acquisition, invasion and immunomodulation [247]. Exosomes developed from *S. mansoni* featured multiple candidates for vaccines, comprising proteins involved in several life cycle phases, suggesting their potential significance at different phases of the parasite’s life cycle [246].

### 4.3. Exosome-Based Vaccines in Clinical Trials

Exosome-based clinical studies can be categorized into three groups, each with a different methodology. Exosomes can first be utilized to transport medications to specified sites. Exosomes are also made from mesenchymal stem cells. Additionally, patient reactions are triggered by exosomes that contain particular mRNAs and miRNAs [231]. In 2005, DEX vaccinations were administered to patients with non-small cell lung cancer (NSCLC) who had HLA A2+, and the results were reported. DEXs containing HLA-restricted melanoma-associated antigen (MAGE) peptides were infused into the patients. All patients responded favorably to the vaccine following the administration of four weekly doses. However, only one-third of the patients demonstrated MAGE-specific T-cell responses, whereas NK-cell activity increased in two of the four patients studied [248]. In the second study, DEXs made from DCs that had been pulsed with MAGE and inoculated were used to administer vaccinations to melanoma patients. Except for five patients out of fifteen who experienced a grade I fever, no patient reported any severe toxicities; nonetheless, no peripheral blood CD4+ and CD8+ cell responses specific to MAGE were seen. It is interesting to note that DEX vaccination also caused NK-cell effector actions, and NK-cell infiltration at the tumor site increased in eight out of thirteen individuals [249]. Exosomes from developed DCs generated more potent T-cell activation, according to earlier studies. The DEXs generated by DCs pulsed with IFN-g were used in a phase II clinical trial in patients with NSCLC. A patient who experienced grade III hepatotoxicity was the only one to experience any harm. DEX vaccination stimulated NK-cell activity rather than T-cell immunity against cancer in this case [250]. A nonrandomized phase I/II clinical trial used exosomes produced by DCs pulsed with SART1, a biomarker for esophageal squamous cell cancer, to develop a vaccine. In addition to DEX vaccines, various exosome-based vaccinations have also been described in clinical trials. Exosomes from ascites (AEXs) and granulocyte-macrophage colony-stimulating factor (GM-CSF) were used in a phase I clinical trial as an immunotherapy for colorectal cancer. All patients were able to endure the four AEX injections every week for colorectal cancer without any problems. A powerful antitumor cytotoxic T-lymphocyte response against the colorectal cancer biomarker carcinoembryonic antigen was observed in advanced colorectal cancer patients treated with AEXs plus GM-CSF [231]. In addition to cancer, exosome-based vaccines have been created to treat various chronic disorders. In a phase II/III clinical experiment, exosomes from umbilical cord MSCs were evaluated in individuals with chronic kidney diseases such as interstitial nephritis and type 1 diabetes [251]. No notable side effects were noted by trial participants either during or after therapy. Exosomes made from MSCs enhanced inflammatory immune activation and overall kidney function. A clinical experiment including healthy participants is currently testing the tolerance and safety of inhaling exosomes made from MSCs as an aerosol (NCT04313647). Another clinical study investigating the utilization of exosomes produced from MSCs as a therapeutic approach to treat macular holes is now underway (NCT03437759) [231].

One of the typical gastrointestinal conditions seen in clinical practice is colorectal cancer (CRC). Malignant ascites can develop when CRC cells enlarge and seed the peritoneal cavity. In clinical practice, patients with malignant ascites always have a poor prognosis. However, therapeutic treatment of CRC patients with ascites may benefit from the extraction of exosomes found in effusions and the activation of immune responses against cancer by exosomes produced in ascites (Aex). Aex is a safe, nontoxic, and tolerable cancer vaccine. According to Shengming Dai et al., Aex from CRC patients can be used to induce anticancer immunity, and adjuvant GM-CSF can significantly boost Aex’s effectiveness. In vivo DTH testing demonstrated that Aex alone is sufficient to induce systemic anti-Aex immunity, indicating that Aex is immunogenic on its own. Aex can activate CD8+ CTLs and may induce antitumor immunity specific to tumor antigens such as carcinoembryonic antigen (CEA). In vivo and in vitro, exosomes generated by heat-stressed CEA-positive tumor cells can both initiate and increase an HLA-A*0201-restricted and CEA-specific CTL response due to their ability to collect HSPs and MHC-I molecules [252]. An exosome-based cancer vaccine, when combined with CpG oligodeoxynucleotides or double-stranded RNA, can improve host immune responses against cancers. In clinical studies for cancer vaccines, GM-CSF has been extensively utilized as an adjuvant and may have potential as a vaccine. Studies have demonstrated that tumor cells that have either been transfected with granulocyte-macrophage colony stimulating factor (GM-CSF) genes, alone or in combination with biodegradable GM-CSF capsules, can induce specific immune responses both in vitro and in vivo [253,254]. To induce CEA-specific CTL responses and HLA-A*0201 restriction, the coadministration of GM-CSF and Aex is more effective than the coadministration of Aex alone, confirming the idea that by encouraging antigen presentation and T-cell activation, the coadministration of Aex and GM-CSF may improve the effectiveness of Aex vaccination. Therefore, Aex with GM-CSF may provide a different option for CRC immunotherapy. With further characterization of Aex, such as the identification of immunosuppressive cytokines for quality control, the precise source of exosomes in Aex, and optimization of the treatment strategy, the clinical effects of Aex-based immunotherapy will be significantly improved [252].

### 4.4. Exosome-Based Vaccine against SARS-CoV-2

Due to the coronavirus outbreak, the priority of exosome-based therapeutic clinical studies has recently switched from addressing cancer to combating COVID-19 in an attempt to design novel vaccines [253,255]. An engineered exosome-based vaccine that contains the spike (S), membrane (M), nucleocapside (N), and envelope proteins (P) of the SARS-CoV-2 virus promotes humoral and cell-mediated immunity as well as long-lasting protection. Strong NAb and T-cell response induction brought on by these vaccinations give extended immunity without running the danger of reverting vaccination-induced virulence and preexisting immunity. Exosomes, which are virus-free, have a better absorption rate and lower antigenicity than currently used vehicles such as adenoviruses or LNPs, which would also satisfy the needs for a perfect vaccine that does not approach booster doses. This need could also be satisfied by combining these immunogens with an effective delivery technique, such as exosomes [235]. Clinical trials have indicated that receptor-binding domain (RBD)-based vaccinations can induce an immune response that can neutralize and protect against SARS-CoV-21. An inhalable SARS-CoV-2 exosome vaccine is composed of recombinant SARS-CoV-2 receptor-binding domains (RBDs) attached to lung-derived exosomes, which are more effective than liposomes in retaining the RBD in the mucus-lined respiratory airway and lung parenchyma. A vaccine administered to mice stimulated mucosal IgA responses, CD4+ and CD8+ T cells that produced Th1-like cytokines, and RBD-specific IgG antibodies. SARS-CoV-2 pseudovirus was not detected in the mice after challenge. The vaccine attenuated severe pneumonia after two doses were administered to hamsters after a live SARS-CoV-2 challenge [254]. Shang-Jui Tsai et al. purified exosomes for their research and loaded them with mRNAs that would allow them to express LSNME, an artificial fusion protein that includes pieces of the viral spike, nucleocapsid, membrane, and envelope proteins, as well as a functional form of spike. The resulting combinatorial vaccine, LSNME/SW1, was administered to male C57BL/6J mice at the age of thirteen weeks, after which humoral and cellular immune responses to the SARS-CoV-2 nucleocapsid and spike proteins were examined, as well as hematological and histological examination to look for any potential side effects in the animals. They discovered that, as would be predicted for a robust response to vaccination, inoculated mice acquired CD4+ and CD8+ T-cell reactivities that respond to both and became visible approximately two months after the vaccination. Additionally, there was a correlation between the spike-reactive CD4+ T-cell response and increased interferon gamma expression, a sign of a Th1 response, and decreased production of interleukin 4, a Th2-associated cytokine. Injection site hypersensitivity, changed white blood cell profiles, or changes in organ shape were not evident in vaccinated mice. In line with these findings, they also found mild but persistent anti-nucleocapsid and anti-spike antibodies in the plasma of animals that had received the vaccination. Overall, these findings support the use of exosomes for delivering functional mRNAs into target cells in vitro and in vivo. More specifically, they demonstrated that LSNME/SW1 vaccination generated widespread immunity to various SARS-CoV-2 proteins [256].

Exosome therapies are being used in 12 ongoing clinical trials at ClinicalTrials.gov. Exosomes overexpressing CD24 at two dosages with a patient follow-up of 23 days are being tested in phase I (NCT04747574) and phase II (NCT04902183) independent clinical trials on patients with mild or severe COVID-19 infection. Currently, two phase I and II clinical trials (NCT04602442 and NCT04276987) are being conducted to determine whether aerosol inhalation of bone marrow MSC-derived exosomes is effective and safe in treating severe SARS-CoV-2 pneumonia and COVID-19 patients within 28 days of the initial treatment. A phase I/II clinical study (NCT04798716) is investigating the efficacy and safety of administering MSC-derived exosomes to severe COVID-19 patients on an ascending dose of 2:4:8 every other day. These clinical trial descriptions suggest that MSC-derived exosomes may lessen pathological impairment and lung inflammation. Only one trial’s findings have been released thus far (NCT04491240), following a 10-day period in which patients inhaled 3 mL of MSC-derived exosomes twice daily, and there have been no reports of any negative side effects. Due to their ability to elicit anti-inflammatory effects and alter immunological responses, MSC-derived exosomes may be useful in the design and development of future COVID-19 vaccines [235].

## 5. Nanoparticle-Based Nucleic Acid Vaccine

Almost all of the vaccinations that are considered to be the safest and highly efficient are those that are centered on attenuated forms of live agents (such as smallpox, measles, oral polio, rubella, and mumps), which culminate in an infection but do not lead to diseases [257]. Conversely, the sudden advent of illnesses such as SARS-CoV-2 and H1N1, as well as rapidly evolving fatal diseases such as Ebola, prove problematic for conventional vaccines that with the usual vaccine production pipeline may require on aggregate over 10 years to produce or, as with Ebola, needing an expedited 5-year development [258,259,260]. As a consequence, many people become ill and die throughout the process of developing, distributing and implementing vaccines. Genetic variation of viral strains, particularly late in the flu season, makes it difficult for traditional seasonal annual vaccine compositions to complement the viruses currently in circulation [258,259,260]. SARS-CoV-2 vaccinations, for example, require a long time to produce and deploy, as proven by the 2009 H1N1 pandemic and the Ebola crises of 2014–2016. This pressing issue necessitates the development of a new vaccination manufacturing methodology. Modern vaccination approaches, including virus-like particles, peptide-based vaccines and nucleic acid-based vaccines, have been developed in recent decades to supersede inactivated or live attenuated vaccines [261]. These latest advancements were designed to increase vaccination persistence, reliability, and affordability [138]. Immune responses kindred to those elicited by live attenuated vaccines can be induced by the use of nucleic acid vaccines. It is possible to fight infection by administering proteins that resemble disease antigens through DNA or messenger RNA (mRNA). DNA and mRNA vaccines attempt to stimulate humoral and cell-mediated immunity through the creation of neutralizing antibodies and cytotoxic T lymphocytes (CTLs) by using the machinery of host cells to generate coded protein antigens. Generic DNA/mRNA production technologies allow for rapid turnaround of vaccines, making it possible to modify sequences in response to evolving disease strains and eliciting both antibody and cytotoxic T-lymphocyte responses. These advantages make nucleic acid vaccines an attractive option for vaccine development [262]. There are several advantages to using this sort of vaccine; they are: (1) safe, since they do not exploit living organisms; (2) effective, because they mimic a live infection by expressing antigens in situ following immunization, triggering both B and T-cell activation (including cytotoxic T cells); (3) targeted, as the immunological responses are tailored exclusively to resist the specific antigens; and (4) a platform technique competent of employing generic manufacturing processes capable of rapid response [257]. Despite these numerous advantages, no human vaccinations using nucleic acids have been approved for human administration thus far, and it appears that existing regimens do not utilize this highly intriguing technique [258,263]. Efficacious distribution to cells remains challenging, which is the primary reason for this. Endogenous nucleases quickly breakdown fragile nucleic acids in the human body. To even reach the cytoplasm (for mRNA vaccinations) and nucleus (for DNA vaccines), DNA/mRNA should indeed transcend various cellular barriers. As a result, vaccinations based on nucleic acids have limited immunogenicity. Finally, because of these factors, nucleic acid vaccines have not yet been clinically tested in people through Phase 3 [264,265].

As viral vector distribution has the potential to escalate public health concerns that might impede wider adoption, nonviral means of gene administration are rapidly being investigated. Nonviral vectors, such as NPs, are widely utilized in diagnostic imaging and medicine administration, among other uses. Intracellular uptake is made easier by the NPs’ small size, which is generally under 200 nm in diameter. Because viruses and NPs share a comparable size range, nanotechnology has the potential to have profound effects on vaccine development. It is possible to use NPs to encapsulate medications that may be delivered to diseased cells [266], and encapsulation using NPs improves the solubility of the medication. In regard to payload delivery systems, NPs are ideal because of their high volume-to-surface-area ratio (V/S), biodegradability, and lower cytotoxicity [267]. Improved medication efficacy is achieved in part by functionalizing the NP surface with targeted moieties while dosage is decreased to achieve optimal therapeutic pharmacokinetics [268]. To release the DNA/mRNA payload and prevent endosomal damage, endolysosome-sensitive nanomaterials have also been constructed and investigated [269]. Since lymph nodes (LNs) contain sizable numbers of subcapsular sinus macrophages, follicular dendritic cells, T cells, and B cells, the conveyance of NPs to LNs has also been investigated in NP design. It can be accomplished by delivering NPs to APCs that move to LNs and by draining smaller NPs 10–100 nm in size to LNs [270]. These formulations can be tailored to code-liver predefined dose combinations of DNA and/or mRNA into each particular target cell for a synergistic immunological impact.

Nucleic acids are difficult to deliver into cells due to their susceptibility toward endogenous nucleases, heavy negative charges on the nucleic acids hinder cell internalization, and the unspecified interferon signal generated by the involvement of foreign nucleic acids inside the cytoplasm, all of which are substantial obstacles to clinical translation [271]. Consequently, an NP nucleic acid delivery mechanism should successfully enclose negatively charged nucleic acids, safeguard against internal enzymes, and promote cellular absorption and intracellular distribution. If the NPs specifically target APCs or LNs, this is an added benefit.

Only a handful of nanomaterial-based nucleic vaccine delivery systems have made it into clinical trials successfully, and none have been given the go-ahead for usage thus far. For the induction of specific humoral and cell-mediated immune responses, nucleic acid vaccine strategies have been found to be effective in human trials, but from a therapeutic point of view, there are still some limitations, as the dose regimen requirement in humans is much higher than the animal doses [272]. Vaxfectin, a cationic liposome that can ionically attach to DNA and enhance the immune response against H5N1 influenza-associated proteins such as HA, nucleoproteins, and viroporins, is a nanomaterial-based adjuvant that has been investigated in a clinical environment [273]. Vaxfectin cannot be considered to be an effective delivery system because it does not enhance the transfection efficiency; hence, it can be considered an adjuvant. It was found that immune responses against influenza A virus and H5N1 were increased by the Vaxfectin-adjuvanted nucleic acid vaccine, as a similar effect was shown by an inactivated protein-based vaccine. As a result, it was considered to be an effective combination for the prevention of pandemic disease [274]. In another study, Vaxfectin was found to be under phase I clinical trials against the tetravalent dengue virus, and it was found to be effective as an enhancer of cell-mediated immunity and safe at the same time [275]. The nucleic acid vaccine strategy was found to be impactful as vaccine therapy for cancers, as it shows minimal adverse effects with excellent immune response-enhancing properties, but it was limited by inherent immunosuppressive properties as well as mutation in the tumor epitope, exhaustion of helper T cells, development of tolerance for antigens, and influx of immunosuppressive cells and tumor-linked macrophages [276]. However, as previously indicated, as opposed to nanomaterial-based delivery techniques, the majority of DNA vaccines that have advanced to clinical trials are administered as naked DNA or through the use of microparticles. For instance, a DNA vaccine expressing the NY-ESO-1 antigen, a frequent biomarker linked to numerous types of cancer, was delivered via gold microparticles [277]. The study demonstrated DNA vaccination as a possible candidate for cancer immunotherapy by demonstrating its ability to trigger antitumor cell-mediated immune responses. In a more recent clinical trial investigation, DNA was administered as an immunotherapy against leukemia using the cationic liposomal adjuvant JVRS-100 [278,279].

## 6. Nanoparticle Uptake and Immunity

The uptake of NPs and immunogenicity are quite closely related. When nanomaterials are introduced into the living system, the body evokes an immune response in the form of inflammation. Having greater insight into the correlation of NPs with the immune system would yield useful information about the possible health benefits and toxicity elicited by the NPs inside the body [36]. Furthermore, a profound understanding of how the immune system responds to these particles is not only crucial for developing various drug delivery systems for immunomodulatory effects but also important for the immunosurveillance of malignant or premalignant cells in the body [280,281].

Cellular uptake into dendritic cells is significantly improved when delivered by using NP as a carrier rather than via the drug molecule alone; in some cases, an uptake increase of 30 times was found to be achieved [282,283]. The interactions between NPs and cells, as well as their physicochemical parameters, have been proven to have a significant impact on cellular uptake. Studies have shown that NPs with smaller particle sizes exhibit higher uptake than larger particles. In terms of morphology, rod-shaped nanoparticles were less easily absorbed by cells than nanospheres and nanostars. Hydrophobic particles have shown enhanced uptake due to lipophilic interactions. In addition, anionic and amine-functionalized particles have been demonstrated to exhibit potential uptake, which has garnered worldwide attention. Additionally, capping of particles significantly improves biological responses due to efficient uptake [284,285,286,287,288]. Table 4 provides a summary of the current research that was undertaken to analyze how these qualities affected the mechanism and effectiveness of cellular uptake.

In addition to the extent to which they are absorbed by the cells, the mechanisms through which NPs enter cells will have a substantial impact on the type of immune response that is elicited [308]. To achieve efficient cellular internalization of NPs, detailed knowledge of the various uptake mechanisms is of paramount importance. As NPs are comparable in size to biomacromolecules, the uptake of NPs is primarily facilitated through the endocytic pathway, which is the major internalization method for biomacromolecules [309,310]. Endocytosis can be classified into phagocytosis (engulfing large molecules by phagocytic cells) and pinocytosis (internalization of fluids by small vesicles). Furthermore, pinocytosis consists of four main subtypes, viz. clathrin-mediated and caveolin-mediated endocytosis, micropinocytosis and clathrin- and caveolin-independent endocytosis. Caveolae/raft-mediated endocytosis occurs due to the interaction of cholesterol-enriched calveolin-mediated membrane receptors with nanomaterials, resulting in the formation of caveolae. Clathrin-mediated endocytosis follows a similar pattern and occurs via the formation of clathrin-coated pits in the cell membrane. Micropinocytosis is the actin-driven nonspecific internalization of extracellular fluids and solute particles [311,312]. Furthermore, the independent pathways involved in internalization do not require clathrin or caveolins for cellular uptake [313]. Additionally, uptake of NPs can be achieved primarily through the use of electroporation and microinjection that can transport NPs forcefully across the membrane barrier. However, despite their significance in cellular uptake, these two techniques exhibit a number of limitations notably related to the deformity of the cell membrane [314].

The uptake mechanism of NPs is also greatly influenced by the physicochemical properties of the particles [315]. Polymeric NPs of size 43 nm, for example, have been reported to be taken up by clathrin-dependent endocytosis, whereas particles of size 43 nm have been found to follow an independent pathway. The potential of macrophages to uptake materials can be significantly impacted by how the macrophage membrane moves under the control of actin, and is regulated by NP morphology. Additionally, in comparison to spherical NPs, rod-shaped particle phagocytosis is frequently limited [281,316,317]. Table 5 includes some of the recent research studies that were conducted to determine the relation between the effect of NP properties and the response obtained from immune cells.

Nanocarriers have a number of advantages over traditional vaccines in regard to the delivery of antigens. One of the major advantages is that NPs are identified as foreign materials by the immune system, leading to an enhanced immune response. Additionally, they increase the amount of time the antigen is exposed to the immune system by causing the formation of an antigen depot that releases antigen gradually [316,317]. It is also possible to tailor nanocarriers to specifically target immune cell receptors and lymphoid tissues so that they can be used to enhance adjuvant and antigen delivery to obtain specific immune responses. The treatment outcome will always be determined by the nanomaterial’s interactions with biological fluids and the resulting immune cell responses. Since NPs are included in vaccine formulations, there is less need for supplemental doses, and a self-adjuvant response is created by the enhanced absorption of particles by antigen-presenting cells [36,328].

## 7. Nanoparticle Vaccine Safety and Regulatory Concerns

Even though a great deal of research has gone into making new delivery platforms focusing on nanotechnology, there are still few nano-based adjuvants in business. Existing research intends to design smart platforms capable of facilitating the detection and conveyance of multiple antigens plus warning signals, primarily to DCs and highly potent APCs [329]. Nanovaccines are believed to augment phagocytic cell activation and maturation by boosting the engagement of antigens with them, which promotes the delivery of processed antigens to T cells. These are in fact extremely adaptable systems that, based upon the route of administration, can stimulate vigorous cellular, humoral, and mucosal immune reactions [330,331].

However, the scientific community, businesses and regulators face significant obstacles in promoting these products’ commercialization as well as in preventing their failure in subsequent phases of clinical testing. Despite the immense and innumerable prospective benefits, exhaustive study is still desired to determine the effects of the distinct physicochemical characteristics and modes of action of nanotechnology-based systems on bioavailability, intracellular signaling, cellular internalization, and cellular uptake [138,332,333]. Table 6 gives a summary of active clinical trials for nanoparticle-based vaccines.

Regarding the safety issues relating to physicochemical attributes and the ensuing interactions with biological systems, precise input should be given to regulators. The recommended route of administration for upcoming clinical usage must be studied, and rigorous toxicology investigations must be conducted for a vaccine lot generated utilizing the raw components and manufacturing techniques that will be used for the final vaccine product. Necessarily, the manufacturer is expected to provide information on local tolerance and repeated dosage toxicology conducted in preclinical studies with at least five male and female animals per batch and time course. It is important to provide information on the animal’s weight, feed intake, local responses, hematological status, and immunology. Additionally, the WHO mandates the reporting of histological evaluations of several tissues, particularly the injection site for vaccines made with novel adjuvants [334].

The potential toxicity of NPs has been a concern with their integration into biomedical settings for a while in part because some materials that are normally thought to be benign change when they are in the form of nanoparticles and can sometimes become hazardous. Owing to their small size, NPs have easy access to numerous body tissues and organs, but they can be a double-edged sword. Injecting the nanovaccine into the skin may produce dermatological issues, consuming it orally may have gastrointestinal effects, and introducing it through the nasal route may provoke respiratory issues. Cardiovascular problems can also be brought on through the parenteral method. Additionally, if these nanomaterials are able to cross the blood-brain barrier, they may harm the brain [335,336]. Based on rat investigations, there is a known risk that the accumulation of NPs may cause vascular thrombosis. The usage of NPs in vaccines, on the other hand, has the potential to cause NPs accumulation in the cell, which raises problems, particularly with regard to long-term exposure. Additionally, there are special problems with the components employed in NPs. Given the multitude of animal vaccines that contain ISCOMs, the safety of saponin-based adjuvants is still unknown, which has up until now prohibited their approval for use in humans. In light of this, there are still reservations over the inclusion of NPs in vaccination [337,338].

**Table 6:** Summary of active clinical trials for nanoparticle-based vaccines.

Biological	Condition	Phase	Sponsor	Reference
EBV gp350-Ferritin Vaccine Matrix-M1	EBV Epstein-Barr Virus Infection Infectious Mononucleosis	Phase 1	National Institute of Allergy and Infectious Diseases	[339]
GBP510 adjuvanted with AS03 (Receptor-Binding Domain (RBD) 25 µg/dose). ChAdOx1-S not less than 2.5 × 10^8^ infectious units.	Covid19	Phase 3	SK Bioscience Co., Ltd., Seongnam-si, Korea	[340]
GBP510 adjuvanted with AS03 (RBD 10 μg/dose, 25 μg/dose; Stage 1 and stage 2) GBP510 (RBD 10 μg/dose, 25 μg/dose; Stage 1 and stage 2) Normal saline (0.9% sodium chloride solution; Stage 1 and stage 2)	COVID-19 (Healthy Volunteers)	Phase 1 Phase 2	SK Bioscience Co., Ltd.	[341]
GBP510 adjuvanted with Alum (RBD 10 μg/dose, RBD 25 μg/dose; Stage 1 and stage 2) Normal saline (0.9% sodium chloride solution; Stage 1 and stage 2)	COVID-19 (Healthy Volunteers)	Phase 1 Phase 2	SK Bioscience Co., Ltd.	[342]
UFluA 20 µg each antigen/dose UFluA 60 µg each antigen/dose Placebo	Human Influenza	Phase 1	Emergent BioSolutions	[343]
NVX-CoV2373	SARS-CoV-2 Infection	Phase 2	Novavax	[344]
SARS-CoV-2 rS/Matrix M1-Adjuvant Placebo Licensed seasonal influenza vaccine	SARS-CoV-2 Infection	Phase 3	Novavax	[345]
SARS-CoV-2 rS/Matrix-M1 Adjuvant and Placebo (Initial Vaccination Period) SARS-CoV-2 rS/Matrix-M1 Adjuvant and Placebo (Crossover Vaccination period) SARS-CoV-2 rS/Matrix-M1 Adjuvant (Booster Vaccination) SARS-CoV-2 rS/Matrix-M1 Adjuvant (Second Booster Vaccination)	SARS-CoV Infection	Phase 3	Novavax	[346]
25 µg SpFN_1B-06-PL + ALFQ (QS21 Adjuvant) Drug: Sodium chloride, USP, for injection (0.9% NaCl) 50 µg SpFN_1B-06-PL + ALFQ (QS21 Adjuvant)	SARS-CoV-2 Infection	Phase 1	U.S. Army Medical Research and Development Command	[347]

## 8. Cytotoxicity of Nanoparticles

Researchers have employed a variety of materials, including VLPs, metals, bioceramics, nonmetals, lipids and polymeric materials, to make nanoparticles with various physical, chemical and electrical parameters that interface uniquely with a selected organ or cell. Conversely, NPs may promptly penetrate lipid bilayers, interface with delicate human organs and have a variety of deleterious ramifications [348]. By means of the mononuclear phagocytic system, biliary clearance and renal urinary system, NPs are removed from the body. Innate immunity is also influenced by metallic nanoparticles. In vitro tests revealed that metallic nanoparticles exhibit cytotoxicity and genotoxicity, as well as the ability to interfere with cytokine production and gene expression due to receptor alterations. The accumulation of oxidized glutathione due to nanoparticles can produce stress, which leads to cell death and cancer [349]. NP physicochemical properties may also promote the uptake of antigen-presenting cells, but they may also have unfavorable physiological consequences on other tissues, such as apoptosis or necrosis. However, it has been demonstrated that intranasal administration of nanoparticles causes lung impairment by triggering mitochondrial stress, which causes the development of inflammatory cytokines and cytotoxic cellular signaling. Another issue with NPs is that they might clump together and obstruct blood arteries in the host. To decrease cytotoxicity, nanoparticle manufacturing can be changed to methods that produce repeatable NPs in terms of size, content and shape [350]. International standard-setting bodies have stated that “size, solubility and surface charge of NPs should be utilized as predictors of nanoparticle toxicity” [351]. Nanomaterials with a size of less than 100 nm can impair the function of distal organs and induce mitochondrial stress and pulmonary inflammation via mechanisms such as free radical generation, hydrophobic interactions and redox reactions. Additionally, unstable NPs can form large micrometer-scale aggregates, which can become entrapped in the capillary bed of the lungs and constitute a major threat to patients [352]. Due to the variety in shape, size, charge, methods of production, surface chemistry, tendency of aggregation and chemical composition, carbon NPs show cytotoxicity [353]. Cell type is the most significant factor that affects the cytotoxicity of carbon nanoparticles. Among all carbon nanoparticles, pristine C60 is a less toxic NP due to its lower cellular uptake and noninducing effects on apoptosis and nitric oxide release [354]. The addition of water-soluble substances decreases the cytotoxicity of nanoparticles. Single-walled carbon nanotubes (SWNTs) show more cytotoxicity than C60 at higher concentrations [355]. To explain the cytotoxicity reported with SWNTs, several possibilities have been proposed. The first is related to the method of manufacture, as the synthesis of SWNTs necessitates the use of metal catalysts, which can be harmful in and of themselves. Decreased glutathione levels, higher oxidative stress and changes in the nucleus and mitochondria are observed at greater doses and longer incubation durations [353]. Another factor associated with the cytotoxicity of NPs is particle aggregation. Wick et al. stated that due to the larger size and stiffness of nanoparticles, agglomerated SWNTs show cytotoxicity [356]. Multiwalled carbon nanotubes (MWNTs) are more dangerous than SWNTs. MWNTs are found in the cytoplasm and near the nucleus and can easily bind to the cell membrane, which may be attributed to their cytotoxic effects [357]. Hydrophobic MWNTs are less hazardous than MWNTs coated with hydroxyl or carboxyl groups. Due to their smaller size, gold NPs can easily enter cells, which leads to cytotoxic effects. Types of surface coating agents also play a significant role in gold NP cytotoxicity [353]. Types of chemicals used to prepare gold nanorods also play an important role in their cytotoxic effects. A minor amount of CTAB-stabilized gold nanorods has been linked to high cytotoxicity. The cytotoxic impact was caused by free CTAB in solution [358]. Quantum dots are one type of NPs from 2 to 100 nm in diameter. Due to the presence of toxic core metals such as lead, cadmium arsenic and selenium, it shows cytotoxic effects. The degradation of these quantum dots can produce free radicals, which can be minimized by coating the core of quantum dots and making it biocompatible [353]. Another factor that plays an important role in the toxicity of NPs is surface charge; neutral surfaces are more biocompatible, while cationic surfaces show more toxicity due to their affinity toward negatively charged cell membranes than anionic surfaces [359]. When NPs enter the human body, they interact with cells and proteins in the blood, which leads to cytotoxicity. The surface binding site, surface charge, metal ion dissolution from nanoparticles, electronic properties of nano oxides and hydrophobicity also influence their cytotoxicity by regulating cellular uptake, oxidative stress, apoptosis, autophagy, and inflammation [360,361]. Copper oxide NPs show cytotoxicity in A549 and HeLa S3 cells by reducing colony forming ability in these cells. Cell death by copper oxide NPs induced through apoptosis increased the breakage of DNA strands in HeLa S3 cells by inducing H_2_O_2_ [362]. Silicon dioxide and zinc oxide NPs exhibit cytotoxicity depending on the concentration, size and types of cells in which they are used, since different cells show different responses toward NPs [363]. Other factors that affect the cytotoxicity of zinc oxide NPs are cellular uptake of different organisms, dissolution, reactive oxygen species formation and induction of inflammatory responses [364]. Surface chemistry is another factor that predominantly affects the cytotoxicity of NPs. For example, NPs coated with cetyltrimethylammonium bromide (CTAB) show more toxicity than PEGylated nanostars [365].

## 9. Manufacturing Challenges

Even considering all of the prospective benefits of nanoparticles, only a few nanoparticle-based pharmaceuticals have obtained clinical licenses. This is due to a number of challenges encountered at various phases of nanoparticle research and fabrication. To fabricate a reliable material with ideal physicochemical and biological properties, the complex nature of NPs as multicomponent 3-D constructions demands a meticulous prototype and technology, an extensive analogous method of analysis and a scaled-up production procedure that can be replicated. Nanomedicines must be investigated thoroughly in clinical trials and in animal models, especially from the perspective of bioavailability, targeting desired areas and potential immunological toxicity. Minor variations in a variety of factors may impact the safety and effectiveness of nanomedicines [366]. Three-dimensional constructions with preferred spatial configurations are important for nanoparticle-based medicine functions. Because of this, a modest alteration in composition or methodology could have a detrimental effect on the intricate combination of the components. To facilitate highly repeatable production methods for nanomedicines, a thorough understanding of the materials through biophysical assessment and pharmacological investigations may be essential. There are numerous challenges that have to be solved, or nanoparticle-based medicine or vaccines must pass through a series of tests, beginning with the precise characterization and successful manufacturing of these sophisticated complexes before they reach the clinic [366]. Particle size and surface area are some of the most important criteria that need to be considered when manufacturing a nanoparticle-based medicine. Since nanoparticles come in a wide range of sizes, the size distribution of the particles is an important criterion when manufacturing a nanoparticle-based medicine. To convey the full benefits, the majority of nanoparticles should be less than 200 nm. The NP size and size distribution must thus be carefully controlled both during small-scale fabrication and throughout larger-scale production techniques [367]. The surface characteristics of NPs are also important factors for their activity and interactions with proteins and cells. The stability of NPs and the opsonization process are influenced by a variety of surface properties, such as charge, functional groups, and hydrophobicity [368,369]. The selection of an appropriate targeted molecule during the fabrication of a nanoparticle-based therapy is another significant phase in maximizing its efficacy and reducing its adverse effects. The relevant nanoparticle characteristics must be ascertained for the desired indication. While moving in one way may address a problem, it frequently leads to another [370]. As there are no in vivo models that can accurately predict the diverse behaviors of the various types of nanoparticles, the synthesis of nanoparticles must only concentrate on scientific findings and extensive preclinical rodent studies [371]. A successful reproducible production process of nanoparticle-based medicine can be achieved by understanding it at early stages of development [372]. Even though the reproducibility of small-scale processes can be achieved easily, for large-scale manufacturing, achieving reproducibility is a constant challenge. Different sterilization approaches, e.g., autoclaving and γ-irradiation, can damage nanomaterials, especially those carrying biological materials [373,374]. The FDA highlights the need to ensure rigorous supervision over the fabrication phase and the challenges in a proposed guideline for liposomal formulations, stating that “liposome drug products are sensitive to changes in manufacturing conditions, including changes in scale.” This should be considered during the stage of development, and significant fabrication variables (such as temperature, shear force, and scale) should be recognized and assessed [375]. Another challenge of nanoparticle-based medicine is the preparation of in situ nanomedicines. Insights into in situ preparation and self-assembly, in which a number of different materials are mixed together to develop a complex for use in human medicine, have been utilized in several nanomedicines [376]. Environmental safety is also a concern when manufacturing nanoparticles. Because airborne NPs disperse as aerosols, the accumulation of such small NPs in lungs can cause pulmonary toxicity. Additionally, NPs have the ability to penetrate the skin barrier, which may cause several skin-related problems [352,377]. In this regard, NPs synthesized exclusively in a wet system may have a significantly lower environmental effect, potentially similar to the production of other liquid medicinal products [366]. Bioavailability, pharmacokinetics, biodistribution, etc., are some important pharmacological criteria that must be taken into consideration when manufacturing nanomedicines, and small changes in these properties may lead to huge differences [366]. Due to the complexity of nanoparticle-based medicine preparation, endotoxin contamination may occur, which may cause an immunological response in patients [378].

## 10. Conclusions

A broad range of NP delivery systems as vaccine carriers or vaccine adjuvants have been utilized, and each present benefits over existing approaches of vaccine delivery. The use of NPs to deliver vaccine components will be advantageous for treatment of various infectious and immunological diseases, as NPs can easily encapsulate target antigens, proteins, peptides, or nucleic acids and provide sustained release or target-specific release of the vaccine payload into immune cells after crossing biological barriers and long-lasting immunological effects. Many of the NPs mentioned in this review are efficient in provoking both cellular and humoral immune responses that would otherwise not be possible with conventional vaccines. Although these NP vehicles may provide exciting prospects for future vaccination strategies, it is also worth noting their potential drawbacks, particularly those associated with cytotoxicity. Since NPs have a comparatively short history in the practice of medicine, they do not have a long-lasting safety profile in human use. However, the recent success of LNP-based COVID-19 vaccines with high effectiveness and even a good safety profile build confidence in the medical community about nanovaccines. Nonetheless, the NP-based vaccine delivery strategy has strong potential as a delivery platform in human infectious diseases and could be adapted for other presently incurable diseases, such as cancer.

## Figures and Tables

**Table 1:** Advantages and disadvantages of different nanoparticles in vaccine delivery.

Types of Nanoparticle Systems	Advantages	Disadvantages	Example	Ref.
Virus-like particles (VLP)	Free of infectious material	Polydispersed particle size	MalariVax against malaria. (contains core proteins of HBV + epitopes of circumsporozoite proteins of *Plasmodium falciparum*)	[43]
	Shielding of immune- modulators prevent off-target effects	Limited encapsidation		
	Self-adjuvant	Lack of reproducibility		
Liposomes	Phospholipids possess intrinsic adjuvant property When modified, is stable in GI fluids	Less stable than polymer particles Limited antigen loading esp. hydrophilic antigens	Mosqurix^®^ against both *Plasmodium* *falciparum* and HBV	[44]
	Can take in both hydrophilic and hydrophobic antigens	Poor stability of GI naked liposomes		
Immunostimulatory complexes (ISCOMs)	Lipophilic antigens is easy encapsulate	Antigens that are hydrophilic are difficult to incorporate	Quilvax-L™, a vaccine for dogs for prevention of canine lyme disease	[45]
	Natural adjuvant	Exert no depot release profile		
	It is biodegradable			
	Built- in adjuvant property of Quil A			
Polymeric	It is biodegradable	Insufficient protection against antigens	Chitosan-DNA nanoparticles displayed effectiveness against infections caused by T.pyogenes	[46]
	Surface properties can be easily modified to improve immunogenicity	Antigens are released prematurely		
	Antigens can be released in targeted sites	Low antigen protection		
Inorganic nanoparticles	Better protection of adsorbed antigens	Nonbiodegradable	Synthetic SNAs (spherical nucleic acids) based nanovaccines for cancer immunotherapy	[47]
	Chances of premature release is less	Poor aqueous solubility		
	Easy surface modification			
Emulsion	Can take in both hydrophilic and hydrophobic antigens	Poor GI stability	Influenza vaccine Prepandrix^®^ and Pandemrix^®^	[48]
	Self-adjuvant	Premature release of antigens		
Lipid nanoparticle	Safe and versatile vehicles for drugs Biodegradable and biocompatible Improve oral drug bioavailability	Low loading efficiency Drug expulsion during storage	Moderna COVID-19 (mRNA-1273) vaccine and BNT162b from Pfizer-BioNTech against SARS-CoV-2 virus.	[49]
Exosomes	Can deliver vaccine to targeted site	Methods for quantification are not sensitive enough.	A phase II NCT04902183 against moderate or severe COVID-19	[50]
	Modification of the cell is possible by transferring active molecules between cells	High cost		

**Table 2:** Overview of viral therapeutics using nanocarrier technology.

Virus	Nanocarrier Platforms	Constituents	Route of Administration	Experimental Outcomes	Advantage	Phase	Ref.
HIV	Peptide-based nanofibrous hydrogel	Methylamino group, DNA, Naphthalene acetic acid-Glycine-Phenylalanine-Tyrosine	Intramuscular, intradermal, and subcutaneous	The level of IFN-γ and IL-4 cytokine was markedly increased	Biocompatible and biosafety	Preclinical	[51]
Merkel Cell Polyomavirus (MCV)	MCV-like particles	Viral proteins 1 (VP1), Polyomavirus capsid proteins	Intramuscular	Anti-MCV antibody seroprevalence was found to be significantly high in mice inoculated with MCV VLPs. Low antibody seroprevalence of cross-reactive antibodies was reported against LPV VLPs and BKV VLPs, nevertheless, providing for 4.4% and 2.6% of the reactivity against MCV VLPs, respectively.	Nontoxic, biodegradable, and biocompatible	Phase I	[52]
Hemorrhagic septicemia virus (HSV)	Polymeric nanoparticles	Poly (lactic-co-glycolic acid)	Mucosal	In exposure to the attenuated viral antigen, the inoculated fish produce anti-VHSV immunoglobulin (Ig), triggering the elements of the humoral immunological response. The anti-VHSV inhibition percentage was significantly increased in immunized groups as compared to a nonvaccinated challenged group	Fabrication is easy, biodegradable, nonimmunogenic, and low toxicity	Preclinical	[53]
Influenza A virus	Gold NPs (AuNPs)	Cytosine-guanine-rich oligonucleotide, AuNPs	Intranasal	An enhancement in anti-M2e serum Ig concentrations was observed when M2e was coupled to AuNPs.	Improved bioavailability and half-life, low toxic, and biocompatible	Preclinical	[54]
Hepatitis B	Polymeric nanoparticles	Poly (lactic-co-glycolic acid), Poly-lactic acid	Intramuscular and pulmonary	Anti-HBsAg antibody concentration of PLA and PLGA was substantially elevated as compared to plain HBsAg	Site-specific targeted drug delivery, biodegradable, nonimmunogenic, and low toxicity	Phase II	[55]
Viral infections	Nanogel	Cationic alginate-poly ethylenimine	Intraperitoneal	Nanogels dramatically augmented anti-OVA IgG1 synthesis but had minimal influence on IgG2a and IgG2b expression. However, IgG isotypes and anti-OVA IgG were enhanced by nanogels in a dose-dependent manner and improved OVA-specific IFN-γ by 60 times.	Nonimmunogenic, highly biocompatible, controlled as well as sustained drug delivery is achieved	Preclinical	[56]
Human papillomavirus (HPV)	Virus-like particles (VLPs)	VLPs, L1, and L2 proteins	---	---	Targeted site specific, biodegradable, and biocompatible.	Marketed	[57]

**Table 3:** The empirical approaches used to characterize NPs are summarized.

Methods	Outcomes	Reference
Atomic force microscopy (AFM)	Rapid interpretation: chemical component, nanoparticle dimensions, and design in 3D modality	[210]
Cryo-electron microscopy (Cryo-TEM)	Explore intricate growth pathways, agglomeration mechanism	[211]
Differential centrifugal sedimentation (DCS)	Size variation and nanoparticle dimensions	[212]
Dynamic light scattering (DLS)	Detecting coalescence, hydrodynamic dimension	[101]
Electron tomography	Analytical data at the molecular level, representation of accurate 3D nanoparticles, images, and video	[213]
Electron diffraction	Investigate order-disorder transition, lattice variables, and crystal component	[214]
Ferromagnetic resonance (FMR)	Nanoparticle dimension, size variable, M values, magnetostatic domain, surface features, shape, and magnetic anisotropic constant	[215]
Fourier transform infrared spectroscopy (FTIR)	Ligand interaction, surface features	[216]
High-resolution transmission electron microscopy (HRTEM)	Differentiate between polycrystalline, amorphous, and monocrystalline nanoparticles.	[217]
Inductively coupled plasma-mass spectrometry (ICP-MS)	Nanoparticle dimension and concentration, size variation, and characterization of the elements	[218]
Liquid TEM	Explore intricate growth pathways, fabrication of crystal structures, and real-time mapping of nanoparticle development	[219]
Low energy ion scattering (LEIS)	Nanoparticle chemical constitution and thickness	[220]
Mossbauer	Thermal unblocking, differentiate between iron oxides, symmetry, surface spins, magnetic anisotropy energy, and oxidation state	[221]
Nanoparticle tracking analysis (NTA)	Size variation and nanoparticle dimensions	[222]
Photoluminescence spectroscopy	Correlation of optical features and structural properties including flaws, composition, and dimensions	[223]
Scanning transmission electron microscopy (STEM)	Investigate the atomic architecture of heterointerfaces, and crystal geometry	[224]
Small-angle X-ray scattering (SAXS)	Size variation, growth kinetics, and size of the particle	[225]
Superparamagnetic relaxometry	Identify and pinpoint NPs with superparamagnetism, and hydrodynamic size variation	[226]
Thermal gravimetric analysis (TGA)	Stabilizers component and weight	[227]
X-ray diffraction (XRD)	Dimension of crystal granules, crystal geometry, and composition	[228]
Liquid Chromatography (LC)	Quantify and identify the lipid nanocarriers (NCs) of LNC-mRNA approved vaccines	[229]
Nuclear magnetic resonance (NMR) spectroscopy	To quantify and learn more about the new lipid excipients’ molecular structure	[229]
Capillary gel electrophoresis	To investigate the glycosylation and disulfide bonds in proteins, molecular weight, nucleic acid, concentration and protein integrity	[230]

**Table 4:** NP physicochemical parameters and their impact on cellular uptake.

Physicochemical Properties	Nanoparticles	Uptake Mechanism	Remarks	Reference
Size	Silver and polystyrene NPs	Polystyrene NPs size 50 nm (PS-50) via clathrin and caveolae-mediated transport; PS-500 via macropinocytosis	Increased uptake in PS-50 due to smaller particle size	[284]
	Carboxylated polystyrene NPs	Endocytosis	Obstruction of 100 nm particles by particles of size 40 nm in a polydisperse sample, increased uptake of smaller sized particles	[289]
	Silica NPs (SNP)	Clathrin-dependent endocytosis	Inhibition of SNP100 uptake in presence of SNP50	[290]
	Cyclosporin A NPs (CsA-NPs)	Endocytosis (<500 nm); lymphatic uptake (>500 nm)	Increased uptake and intestinal absorption of 280 nm sized CsA-NPs than 522 nm and 2967 nm	[291]
	Coumarin-6-polylactic-co-glycolic acid NPs	Receptor-mediated endocytosis and phagocytosis	Increased uptake of NPs in the size range of 100–200 nm (optimum size) as compared to 50 nm, 500 nm and 1000 nm	[292]
Shape	Mesoporous silica NPs	Endocytosis	Increased uptake and phototoxicity in cancer cells of spherical shaped particles than the rod-shaped particles	[293]
	DNA aptamer AS1411 gold NPs	Passive diffusion	Increased uptake in nanostars as compared to nanospheres in cancer cells	[285]
	Gold NPs	Clathrin-mediated/caveolae-mediated endocytosis	Increased uptake of Au nanospheres than the corresponding Au nanostars	[294]
	DNA-origami shaped designer NPs	Endocytosis	Larger particles with moderate aspect ratio (width: height) showed increased uptake than elongated particles with high aspect ratio	[295]
	Methyl-polyethylene glycol-gold NPs	Clathrin/caveolae-mediated uptake and lipid raft-mediated endocytosis	Increased uptake in stars, intermediate in rods, and least in triangles	[296]
Hydrophobicity	Amphiphilic nanogels	Passive transport	Hydrophobic NPs displayed increased uptake and protein binding in THP-1 cells	[297]
	Lutein stevioside NP	Passive diffusion/clathrin-mediated endocytosis/caveolae/lipid raft dependent endocytosis	Entrapment of lutein in the hydrophobic core of stevioside caused increased uptake and bioavailability of lutein	[298]
	Star shaped polymer-imidaclothiz NP	Clathrin-mediated endocytosis	Entrapment of imidaclothiz in the lipophilic core of star polymer, increased plant uptake of imidaclothiz	[286]
	Liposomal silk fibroin and sodium alginate (https://www.sciencedirect.com/topics/chemical-engineering/sodium-alginate)-dimethylcurcumin NP	Endocytosis	Combination of silk fibroin and sodium alginate (https://www.sciencedirect.com/topics/chemical-engineering/sodium-alginate) provide a platform for loading poorly water-soluble dimethylcurcumin (anticancer activity) through hydrophobic interactions	[299]
	Propranolol-chitosan nanogels	Endocytic uptake	Hydrophobic drug reduced size of nanogel with increased uptake	[300]
Surface chemistry	Anionic polystyrene NPs	Clathrin-mediated medocytosis and micropinocytosis (cations); clathrin-mediated and lipid raft-dependent internalization (anions)	Increased uptake of anionic particles due to cleavage of heparan sulfate from cell surface	[301]
	Ultrasmall superparamagnetic iron oxide NPs (USPION)	Endocytosis	Increased uptake of amine-functionalized USPION as compared to carboxyl-functionalized USPION	[287]
	Superparamagnetic iron-oxide NPs (SPIONs)	lysosomal pathway	Increased uptake of aminated SPIONs and as compared to polyethylene glycolated SPIONS	[302]
	Mesoporous silica NPs and up/downconverting NPs	Endocytosis	Increased uptake of negatively charged particles in acidic pH and conversion to positive charge	[303]
	Thermosensitive liposomal NPs with surface polyethylene glycol/phospholipid 1,2-dipalmitoyl-*sn*-glycero-3-phosphodiglycerol (DPPG_2_)	Endocytosis	DPPG_2_ functionalized anionic NPs exhibited increased uptake of doxorubicin in cancer cells	[304]
Capping	Gold NPs	Clathrin-mediated/caveolae-mediated endocytosis	11-mercaptoundecanoic acid (MUA)-coated particles were more greatly internalized relative to citrate-coated particles	[294]
	Gold NPs	Clathrin-mediated/caveolae-mediated endocytosis	Carboxyl-polyethylene glycol-thiol (PEG) capped particles showed increased uptake in cancer cells	[305]
	Gold NPs	Endocytosis	Increased uptake of Au released from sodium warfarin capped particles	[288]
	Zinc oxide NPs	Endocytosis	L-cysteine capped particles increased uptake in cancer cells	[306]
	Gold NPs	Transcytosis	Increased uptake caused by albumin-capped particles	[307]

**Table 5:** Effect of physicochemical properties of NPs on immunogenicity.

Physicochemical Properties	Nanoparticles	Response of Immune System	Reference
Size	NPs	Regulation of innate immunity (activation of Kupffer cells and B cells) increased in large (100–250 nm) and decreased in medium (10–100 nm) sized NPs	[318]
	Silver, silica, titanium, and magnetic NPs	Increased IL-8 and IL-1β cytokine secretion by all NPs except silver NPs	[319]
Shape	Gold NPs	Increased immune response and IgG production in spherical and star shaped	[320]
	Polyethylene glycol gold NPs	Nanorods increased inflammasome activation	[321]
Hydrophobicity	Synthetic-HDL NPs	Increased immune response of hydrophobic liver X receptor agonist	[322]
	SARS-CoV-2 spike protein B-cell epitome (S461-493)-gold NPs	Increased IgG responses when compared to sole S461-493 with increased released of cytokines	[323]
Coating	Cisplatin -ovalbumin-coated iron oxide NPs	Increased macrophage polarization and proinflammatory cytokines	[324]
	Polyethylene glycol coated NPs	Increased lymphatic transport and modulate immune response	[325]
Surface charge	Silica NPs	Amine functionalized particles decreased monocyte/macrophages activation (nitrite secretion, CD40/CD80, and pro-inflammatory cytokines)	[326]
	Silica NPs	Hydrocarbon groups at the surface formed albumin rich corona, increased pro-inflammatory cytokines (IL6 and TNFα)	[327]

## Data Availability

Not applicable.
